# Editorial: Protein regulation by lipids

**DOI:** 10.3389/fmolb.2025.1669083

**Published:** 2025-08-12

**Authors:** Mikhail V. Bogdanov, Elena G. Govorunova

**Affiliations:** Department of Biochemistry and Molecular Biology, Center for Membrane Biology, The University of Texas Health Science Center at Houston McGovern Medical School, Houston, TX, United States

**Keywords:** lipids, proteins, regulation, membrane, interactions

Lipids provide the complex hydrophobic-hydrophilic solvent within which membrane proteins fold and function, and they act in a more specific manner in determining the folding, final membrane protein organization, and orientation in the membrane. Membrane proteins could interact with membrane lipids in multiple ways, including: (i) direct interaction inside the protein with non-annular or structural lipids acting as co-factors or ligands (ii) close interaction with the first shell of annular lipids which is less specific since involved in regulation of protein conformation via changing general physical membrane properties around the protein and (iii) guiding of proteins into lipid domains (rafts). Lipids may also act transiently in a manner analogous to molecular chaperones in the folding of proteins independently from the maintenance of their final structure. The large number of different membrane lipids with varying structures and properties, along with the characteristic lipid composition of different membrane types, suggests that distinct lipids have specific functions within the membrane. The roles lipids play in cellular processes are as diverse as the chemical structures of lipids found throughout nature. All biological membranes contain a spectrum of lipid enantiomeric and diastereomeric species with diverse molecular shapes and differing in the number of acyl chain length, unsaturation, head and backbone group composition, chirality, ionization, and chelating properties. A delicate balance between these molecular shapes and phase properties is necessary to achieve a functional membrane. Defining lipid function is a challenging task due to the diversity of chemical and physical properties of lipids, as well as the fact that each lipid type is potentially involved at various levels of cellular function. The scope of this Research Topic encompasses both novel findings that report on all aspects of lipid regulation of membrane proteins, as well as the broader implications of these findings. This Research Topic comprises three Original Research papers, two Reviews, and one Hypothesis and Theory.

Crystallographic data provide vital clues into lipid-protein interactions. Haloarchaeal rhodopsins convert solar energy into ionic gradients on the cell membrane or act as light sensors to drive photomotility behavior, and are essential models to study protein regulation by lipids. For structural studies, membrane proteins are solubilized from the native membranes using detergents and then reconstituted in synthetic lipids. Bukhdruker et al. analyzed the highest-quality crystallographic structures of haloarchaeal rhodopsins to investigate the direct molecular interactions between lipid hydrocarbon chains and the hydrophobic protein surface. The authors found that most structures obtained by different crystallization methods still contained tightly associated archaeal lipids, the shape of which closely matched grooves of the protein surface. These findings support the notion that the so-called annular lipids form the nearest layer of hydrophobic lipid side chains, which strongly interact with the hydrophobic protein surface and adopt the shape (straight and bent) of the preferred low-energy conformation, complementary to the given protein structure. An alternative approach to producing haloarchaeal rhodopsins for crystallographic studies is the expression of their genes in bacterial hosts (mostly *Escherichia coli*). Bacterial membranes differ from archaeal membranes in their lipid composition, and some haloarchaeal rhodopsin structures purified from bacterial hosts revealed non-native oligomeric compositions due to the lack of lipids that mediate the contact between protomers in native membranes. In contrast, other structures show bacterial lipids trying to fill in the same crevices in which native lipids are found. Such “foreign” lipid molecules could occupy protein sites, either like or unlike those in the native membrane, and therefore affect packing constraints and/or force the protein protomers to adopt incorrect conformations. The authors also discuss the use of noble gases to probe lipid-protein interactions and provide recommendations for enhancing the informativeness of structural studies.

Super-resolution microscopy is another powerful approach to study lipid-protein interactions. Compartmentalization of different biochemical, biosynthetic, and signaling processes plays a fundamental role in cell function. Over the last decade, this view has been extended from subcellular organelles to single membranes and their primary components, including lipids and proteins. Super-resolution microscopy allows researchers to visualize single protein molecules and their clusters with nanoscale precision, surpassing the diffraction limit of conventional light microscopy. Super-resolution microscopy enables the study of membrane organization, membrane-bound processes, channels, and transporter dynamics at the nanoscale in living cells, coupling imaging with quantitative analysis. Diaz and Arnspang explored the pros and cons of using different techniques (summarized in their excellent Table 1), which allow visualization of single or clustered protein molecules and their organization within cell membranes, reveal how proteins or lipids organize into nanoscale domains, and how these domains change over time. These technologies have shown that membrane proteins and lipids are not randomly distributed but form dynamic, functional microdomains. Significantly, lipids can influence protein function not only through direct interactions but also by modulating other regulatory proteins.

This regulatory role of lipids is particularly evident in pathophysiological processes such as hypertension. Lipids may regulate protein activity not directly, but by acting on other regulatory proteins that modulate the activity in question. Hypertension among children and adolescents has notably increased over the past 2 decades and is associated with an increased risk of stroke, myocardial infarction, and congestive heart failure. Protein kinases C (PKC) regulate the activity of several proteins responsible for blood pressure control. Diacylglycerols (DAGs) activate specific isoforms of PKC and thus are an essential target for pharmaceutical intervention to normalize blood pressure. Alpha-1 antitrypsin (AAT) is a multifunctional protein with anti-inflammatory and protective effects. Its administration has been shown to prevent type 1 diabetes and lower blood pressure in diabetic mice, but the underlying biochemical mechanisms have not been thoroughly studied. Dogan et al. investigated the consequences of pharmaceutical-grade human AAT alpha-1 antitrypsin (hAAT) administration to reduce blood pressure in juvenile salt-induced hypertensive mice. They found that hAAT administration decreased the concentrations of DAGs and other bioactive lipids, attenuated PKC activity, and reduced surface expression of the sodium-potassium-chloride cotransporter (NKCC2) in kidney cortex membrane fractions. These findings suggest that the mitigation of salt-induced hypertension by hAAT administration results from reduced sodium reabsorption in the kidney.

Lipid-protein interactions are also crucial in bacterial pathogenesis. Peripheral membrane proteins associate with the lipid bilayer through their membrane-targeting domains, which can directly interact with the outer head groups or the internal hydrocarbon backbones of the membrane lipids. Oleate hydratase (OhyA) plays a key role in bacterial pathogenesis by neutralizing host antimicrobial fatty acids. This enzyme forms oligomers on membrane surfaces, stabilized by both protein-protein and protein-lipid interactions. Lathram et al. used fluorescence correlation spectroscopy to investigate the impact of membrane curvature and lipid availability on the binding of GFP-tagged OhyA from *Staphylococcus aureus* to phosphatidylglycerol unilamellar vesicles. They found a moderate preference for binding to large vesicles, indicating that membrane curvature plays a primary role in protein binding, as it favors binding to large vesicles over small ones. Lipid-mediated protein binding drives initial contact of OhyA with bacterial membranes, while the protein:lipid molar ratio determines sustained interactions. Phosphorus-31 (31P) NMR spectroscopy revealed two distinct binding modes at low and high protein concentrations. These results suggest that lipids play a dual role as structural stabilizers for the membrane binding of individual protomers and as facilitators of protein oligomerization.

This dual role of lipids is also evident when we consider interactions between amphipathic, peripherally membrane-bound proteins, which primarily associate with lipids through electrostatic and hydrophobic interactions, and sometimes through covalent lipid anchors. Membranes containing preexisting integral membrane proteins may create a complex ternary protein-lipid-protein system, as designated by Rostovtseva et al. The authors designed, investigated, and reviewed this intriguing system, which acts either as a “romantic triad” or a “love triangle” for the first time. The interaction of the amphipathic protein with the membrane can potentially induce conformational changes in resident integral proteins by altering the physical constraints of the lipid bilayer, thereby affecting their function. Alternatively, distortions of the lipid bilayer by embedded integral membrane proteins can create a favorable environment and favorable binding sites within the membrane itself for the docking of amphipathic membrane proteins due to local changes in the lipid composition, geometry, and dynamics of the otherwise “silent” bilayer.

Bilayer dynamics are also a crucial indicator of pathological states, such as ferroptosis. Ferroptosis is a newer form of cell death driven by iron-dependent lipid peroxidation of polyunsaturated fatty acids (PUFAs). Shabanpour et al. computed ferroptotic membrane by virtual replacement of PUFA-containing phospholipids of the native red blood cell membrane with their hydroperoxide counterparts and investigated the effect of observed the biophysical membrane changes (increase in area per lipid, increase in the width and decrease in lipid packing order) on the uptake of doxorubicin, a potent anticancer agent linked to ferroptosis. Umbrella sampling simulations allowed authors to calculate the potential of mean force for the translocation of doxorubicin across two modeled membranes. Surprisingly, the permeability of the ferroptotic membrane was almost ten orders of magnitude higher than that of the native membrane. Discovered ferroptosis-induced membrane “weakening” can be utilized in predicting responses to chemotherapy.

We are still far away from a complete understanding of native-like protein-lipid interactions and the mechanisms by which protein and lipid permanent or transient “fingerprinting” contribute to function. A dynamic view of lipid and protein organization reveals previously unrecognized possibilities for membrane protein function and regulation.

